# Genetic and Physiological Analysis of Iron Biofortification in Maize Kernels

**DOI:** 10.1371/journal.pone.0020429

**Published:** 2011-06-08

**Authors:** Mercy G. Lung'aho, Angela M. Mwaniki, Stephen J. Szalma, Jonathan J. Hart, Michael A. Rutzke, Leon V. Kochian, Raymond P. Glahn, Owen A. Hoekenga

**Affiliations:** 1 USDA-ARS, Robert W. Holley Center for Agriculture and Health, Cornell University, Ithaca, New York, United States of America; 2 Department of Food Science, Cornell University, Ithaca, New York, United States of America; 3 USDA-ARS, Plant Science Research Unit, Raleigh, North Carolina, United States of America; University of Melbourne, Australia

## Abstract

**Background:**

Maize is a major cereal crop widely consumed in developing countries, which have a high prevalence of iron (Fe) deficiency anemia. The major cause of Fe deficiency in these countries is inadequate intake of bioavailable Fe, where poverty is a major factor. Therefore, biofortification of maize by increasing Fe concentration and or bioavailability has great potential to alleviate this deficiency. Maize is also a model system for genomic research and thus allows the opportunity for gene discovery. Here we describe an integrated genetic and physiological analysis of Fe nutrition in maize kernels, to identify loci that influence grain Fe concentration and bioavailability.

**Methodology:**

Quantitative trait locus (QTL) analysis was used to dissect grain Fe concentration (FeGC) and Fe bioavailability (FeGB) from the **I**ntermated **B**73 × **M**o17 (**IBM**) recombinant inbred (RI) population. FeGC was determined by ion coupled argon plasma emission spectroscopy (ICP). FeGB was determined by an *in vitro* digestion/Caco-2 cell line bioassay.

**Conclusions:**

Three modest QTL for FeGC were detected, in spite of high heritability. This suggests that FeGC is controlled by many small QTL, which may make it a challenging trait to improve by marker assisted breeding. Ten QTL for FeGB were identified and explained 54% of the variance observed in samples from a single year/location. Three of the largest FeGB QTL were isolated in sister derived lines and their effect was observed in three subsequent seasons in New York. Single season evaluations were also made at six other sites around North America, suggesting the enhancement of FeGB was not specific to our farm site. FeGB was not correlated with FeGC or phytic acid, suggesting that novel regulators of Fe nutrition are responsible for the differences observed. Our results indicate that iron biofortification of maize grain is achievable using specialized phenotyping tools and conventional plant breeding techniques.

## Introduction

Iron (Fe) deficiency is a worldwide problem that is directly correlated with poverty and food insecurity. Approximately 1/3 of the world's population suffers from Fe deficiency-induced anemia, 80 percent of which are in developing countries [1]. The consequences of Fe deficiency include increased mortality and morbidity rates, diminished cognitive abilities of children, and reduced labor productivity that in turn stagnates national development [2]. The developed world has made tremendous success in alleviating micronutrient deficiencies through dietary diversification, processed food fortification, improved public health care and supplementation. In developing countries, these strategies are often too expensive and difficult to sustain. The major causes of Fe deficiency are inadequate Fe intake/availability from foods and blood loss or increased demand due to disease (e.g. malaria, HIV/AIDS) [3]–[5]. Inadequate nutrition is the more common cause for Fe deficiency and is largely due to poverty, which limits the consumer's dietary choices and thus the quality and quantity of foods consumed [6]. About 75 percent of the world's poor households live in rural areas and the majority are small-scale farmers [7]. The resource-poor typically consume what they grow and are dependent upon a small number of staple crops for the vast majority of their nutrition [8], [9]. This limits the feasibility of processed food fortification as a micronutrient deficiency-alleviating tool for this group and emphasizes the importance of plant-based agricultural solutions for human nutrition problems.

Fe is less available for absorption into the human body from vegetarian as opposed to non-vegetarian diets [10]. The influence of biochemical factors on Fe availability depends on the form of Fe. Iron in plants exists primarily as non-heme Fe. Compounds in food influence non-heme Fe bioavailability by either limiting solubility, or by inhibiting Fe accessibility to the Fe transporter on the intestinal surface; therefore an increase in Fe concentration alone may not solve dietary Fe deficiency problems [11]. Ascorbic acid, cysteine, and the “meat factor” are all compounds that are known to enhance non-heme Fe absorption in the human gut [12]. The primary characterized inhibitors of Fe bioavailability in plant foods are phytate and polyphenolic compounds, although other compounds may also exist [12].

Given the high cost of quantifying Fe bioavailability via human and animal studies, *in vitro* screening of food samples represents the most feasible system for screening large numbers of samples to identify factors and interactions that affect Fe bioavailability [13]. The current state of the art for *in vitro* screening involves a simulated gastric and intestinal digestion of food coupled with measurement of Fe uptake by human intestinal epithelial cells, specifically the Caco-2 cell line [14]. This cell line exhibits the characteristics of small intestine epithelial cells, which is believed to be the primary site for Fe absorption in the human gastrointestinal tract. Caco-2 cells have been shown to exhibit a broad range of morphological and functional characteristics of intestinal epithelia in regards to the uptake of Fe and other nutrients, which make Caco-2 cells an excellent model system [14], [15]. These characteristics include: 1) Caco-2 cells reduce Fe3^+^ to Fe^2+^ via the apical Fe uptake pathway and tightly regulate ferritin synthesis and transepithelial Fe transport within a narrow margin of intracellular Fe concentrations [16]. 2) Transport of Fe in the Caco-2 cell line responds to the Fe status of the cell, as Fe-deficient cells exhibit increased and Fe-loaded cells exhibit decreased transport into the basolateral side of the cells [17]. 3) Factors that inhibit Fe availability (e.g. phytate, polyphenols) and promote Fe availability (e.g. cysteine, β-carotene) have similar effects on Fe uptake into Caco-2 cells as they do in human or animal subjects [18]–[20]. In addition, a comparison study using both human subjects and the Caco-2 cell system concluded that Caco-2 cells predict Fe bioavailability quite well [21].

Cereals make the bulk of the household diets in developing countries and hence are an ideal tool for Fe biofortification. The conventional approach to cereal mineral biofortification has been to work at three levels. These are to increase the density of the mineral nutrient of interest, to decrease the density of anti-nutritive compounds (nutrient inhibitors), and to increase the density of compounds that enhance bioavailability of the specific nutrient. The best example from conventional breeding is a study from the International Rice Research Institute (IRRI), where a new rice variety was developed with substantially more Fe concentration than varieties typically consumed in Asia. A high Fe variety chosen for a feeding study contained 2.6 µg g^−1^ DW more Fe than a standard commercially available rice variety. A nine month, double-blind human study carried out on 192 subjects showed that eating this high Fe rice led to a 17% increase in total body Fe, as measured by serum ferritin and total Fe stores [22]. Rice has also been altered using transgenes to increase Fe bioavailability. One effort used an endosperm-specific promoter to drive the expression of a ferritin gene from *Phaseolus vulgaris,* as well as expression of a thermo-tolerant phytase from *Aspergillus fumigatus* and an endogenous Cys-rich metallothionein-like protein [23]. This triple transgene combination increased the rice grain Fe concentration by up to two fold, while also increasing phytase activity and Cys concentration in the rice grain. However, no test of Fe bioavailability was made, such that the efficacy of this approach for biofortification cannot be evaluated. In a third study, Drakakaki and co-workers (2005) generated transgenic maize expressing both an *Aspergillus* phytase and soybean ferritin in the kernel. In the most active transgenic line, up to 95 percent of the phytate was degraded and a 50% increase in the Fe concentration of the grain was observed. Fe bioavailability was evaluated using the *in vitro* digestion/Caco-2 cell model and demonstrated that phytase expression was directly correlated with Fe bioavailability and uptake [24]. Thus, it is possible to positively impact human nutrition by reducing Fe malnutrition via crop biofortification.

In the current study we used an integrated genetic, physiological and biochemical strategy to begin to understand the determinants of Fe nutrition for humans in maize kernels. The Intermated B73 × Mo17 (IBM) recombinant inbred (RI) population of maize was employed as our study system [25]. The IBM population is a powerful resource for the analysis of quantitative traits and is the community standard for genetic mapping in maize [26]–[27]. We collected two datasets related to Fe nutrition – total Fe concentration in the grain (FeGC) and the bioavailable fraction of Fe in the grain (FeGB), which was measured indirectly via Fe uptake and subsequent ferritin production in Caco-2 cell cultures. These data were then analyzed to identify quantitative trait loci (QTL) that contribute to these traits. Candidate QTL for FeGB were isolated in new varieties to confirm the genetic analysis and provide more convenient research tools. These new varieties have been grown repeatedly in NY and have given significantly different outcomes for FeGB, confirming the validity of the FeGB QTL model. These stocks have also been evaluated outside of NY and produced significant outcomes, indicating that the enhancement of FeGB is not specific to field sites in NY.

## Results

### Analysis of grain iron concentration (FeGC)

**Figure 1:**
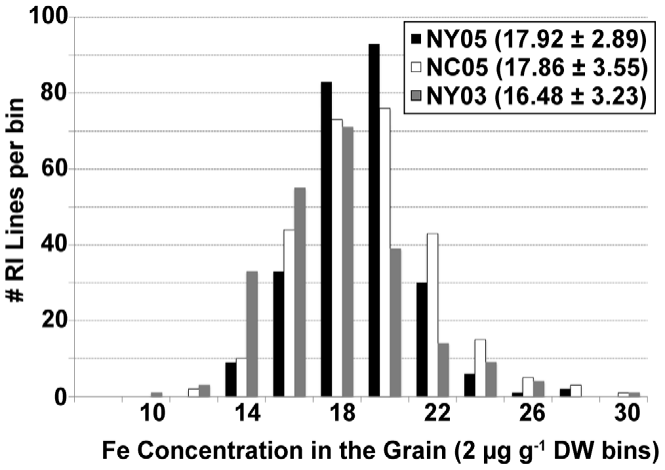
FeGC observed for a maize population. The Intermated B73 × Mo17 recombinant inbred (RI) mapping population was grown in Aurora NY and Clayton NC on research farms owned by Cornell University and North Carolina State University, respectively. Grain Fe concentrations were determined by ion coupled argon plasma emission spectroscopy. The results for the RI lines are organized into bins of 2 µg Fe g^−1^ grain DW for the histogram. Median population values are reported along with standard deviations for each of the three contributing data sets.

Grain Fe concentration (FeGC) was the first parameter used to estimate the nutritional quality of grains in the IBM RI population. This mapping population was grown twice in two different field seasons in NY and once in NC in replicated trials. An analysis of variance indicated that the RI Line was the greatest contributor to variance in the FeGC trait, suggesting that strong genetic control for the trait exists (Table 1). In fact, heritability was estimated at 0.745, confirming this observation. However, significant variance was also found that was due to site and year, such that environmental and random factors also influence the FeGC trait. Examining the average values for each RI line, highly similar values were observed for the FeGC trait from three year/site replicates (Figure 1). Transgressive segregation was observed consistently, as both B73 and Mo17 parents fell close to the median value for the population. The range of variation from minimum to maximum values was somewhat limited, only on the order of 3-fold.

**Table 1:** Analysis of variance for grain iron concentration (FeGC).

Source	DF	Sum of Squares	F-score (GLM)	p-value (GLM)	%Variance (REML)
Line	224	4810.25	2.79	<0.0001	23.46
Year [Site]	1	232.96	30.28	<0.0001	6.29
Site	1	121.50	15.79	<0.0001	-2.67
Error	1034	7956.50			
Total for Model	1260	13046.61	2.93	<0.0001	100.00

General Linear Model (GLM) and Restricted Maximum Likelihood (REML) analyses of variance (ANOVA) were used to describe the variance in grain iron concentration due to Line, Year (nested within Site), and Site terms from the NY05, NY03 and NC05 data. Heritability (h^2^_b_) was estimated at 0.745.

To account for the contribution of genetic and environmental factors to the FeGC trait, we estimated the best linear unbiased predictors (BLUPs) for each RI line to facilitate quantitative trait locus (QTL) detection across the three data sets. Composite interval mapping analysis on the BLUPs identified three modest QTL for FeGC (Table 2). Two of the superior alleles were donated by the Mo17 parent (FeGC-5.1 and FeGC-9.1), while the third came from B73 (FeGC-2.1). This pattern of both parents donating superior alleles is consistent with the observed transgressive segregation. A multiple interval model for these QTL indicated that approximately 26% of the phenotypic variation was due to these three factors. Single marker analysis was also used to identify QTL using more permissive rules. However, these QTL failed to explain substantially more variance and thus are not reported here.

**Table 2:** Locations of FeGC QTL detected by composite interval mapping analysis from summary trait data.

Trait-Chr. Donor	Peak Location (cM)	Closest Marker	LOD Score	Additive Effect	R^2^	CI (Peak -1 LOD)	CI (Peak -2LOD)
FeGC-2.1 B73	194.11	MMP144	6.21	+0.415	0.101	188…200	176…202
FeGC-5.1 Mo17	279.11	RZ87	7.694	-0.447	0.12	276…285	273…285
FeGC-9.1 Mo17	77.11	SH1	4.61	-0.39	0.093	69…85	67…89
FeGC	MIM model				0.261		

BLUPs were estimated from the analysis of variance and used as summaries for quantitative trait locus detection by composite interval mapping. Confidence intervals (CI) for each QTL are reported at two different confidence values. Genetic locations refer to IBM v1 map coordinates. Positive values for the additive effect denote B73 provided the superior allele. Multiple Interval Mapping (MIM) was used to estimate the 3-factor model.

### Analysis of grain iron bioavailability (FeGB)

**Figure 2:**
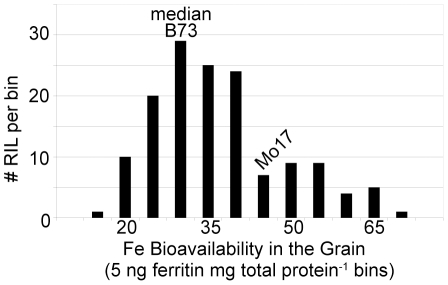
FeGB observed for a maize population. The Intermated B73 × Mo17 RI population was grown at Aurora NY in 2003. Grain samples were evaluated for grain bioavailable Fe via a Caco2 cell culture bioassay. The bioassay measures the amount of ferritin storage protein produced in the human cells in response to the maize samples, and thus estimates how much Fe was absorbed from the samples. Results for the RI lines are organized into bins of 5 ng ferritin mg total protein^−1^ for the histogram.

Grain iron bioavailability (FeGB) was the second parameter used to estimate the nutritional quality of grains in the IBM RI population (Figure 2). Due to the complexity of the Caco-2 bioassay (i.e. that 145 RI lines required 6 person/months worth of effort), the 2003 NY field season (hereafter, NY03) series of samples were chosen used for FeGB phenotyping to generate the data necessary for QTL mapping. Maize seed Fe bioavailability had more than twice as wide a distribution as Fe concentration, with a 7.2-fold range from the minimum (8.7 ng ferritin produced by Caco-2 cells mg^-1^ total protein) to maximum values (63.0 ng ferritin mg^−1^ total protein; Figure 2). The population median was 27.3 ng ferritin mg^−1^ total protein among the 145 RI lines sampled from the NY03 field season. The B73 parent was again close to the population median, while the Mo17 parent exhibited greater grain Fe bioavailability. Transgressive segregation was observed for FeGB, as was the case for FeGC, as the range of phenotypes observed was larger than that in the parental varieties. These results indicate that both B73 and Mo17 carry alleles of possible utility for the improvement of grain Fe nutrition.

QTL analysis was first performed for the FeGB trait using Composite Interval Mapping (Table 3). Three modest QTL were detected, where much like FeGC the combination of donors was consistent with the observed transgressive segregation. QTL analysis was repeated using the GLM Select procedure in SAS. While this is a single marker regression analysis, we considered the marker density in the IBM population to be sufficiently dense to counteract any loss of power. Ten significant markers were identified that explained 54% of the variance observed in FeGB (Table 4). This suggests that FeGB may be a more simply inherited trait than FeGC, as a greater number of larger QTL were detected for FeGB than FeGC. Of all the putative QTL detected, there was only one case where FeGC and FeGB QTL were closely located on the maize genome (FeGC-9.1 from Table 2 and FeGB-9.2 from Table 4).

**Table 3:** Locations of FeGB QTL detected by composite interval mapping analysis for 2003 field season.

Trait-Chr. Donor	Peak Location (cM)	Closest Marker	LOD Score	Additive Effect	R^2^	CI (Peak -1 LOD)	CI (Peak -2 LOD)
FeGB-3.1 Mo17	189.2	PSR754B	3.54	-7.96	0.078	185…190	183…192
FeGB-6.1 B73	70.4	PHP20528	5.82	+10.39	0.135	63…74	58…81
FeGB-9.1 B73	377.6	UMC2134	3.70	+9.28	0.103	369…384	367…384
FeGB	MIM model				0.250		

Standardized ferritin protein production values were used for FeGB quantitative trait locus detection by composite interval mapping. Confidence intervals (CI) for each QTL are reported at two different confidence values. Genetic locations refer to IBM v1 map coordinates. Positive values for the additive effect denote B73 provided the superior allele. Multiple Interval Mapping (MIM) was used to estimate the 3-factor model.

**Table 4:** Locations of FeGB QTL detected by GLM Select analysis for 2003 NY field season.

Factor	AIC	F-score	p-value	t-value	Position	Trait-Chr
Intercept	968.18	0	1	5.45	–	–
php20528	941.66	16.57	<0.0001	-5.75	6; 70	FeGB-6.1
csu471	929.75	12.78	0.0005	3.84	9; 102	FeGB-9.2
psr754b	920.46	10.73	0.0013	4.83	3; 185	FeGB-3.1
umc2134	909.61	12.34	0.0006	-4.24	9; 379	FeGB-9.1
umc1910	898.93	12.72	0.0005	4.74	8; 215	FeGB-8.1
umc63a	891.70	11.90	0.0007	-3.68	3; 573	FeGB-3.2
umc1634	884.01	8.19	0.0049	-4.86	9; 179	FeGB-9.3
psr547	877.40	8.82	0.0035	3.56	9; 263	FeGB-9.4
umc23a	870.84	10.34	0.0016	4.38	1; 600	FeGB-1.1
umc1072	863.80	8.50	0.0042	-3.26	5; 540	FeGB-5.1

Source	DF	F-score	p-value	SS		Adjusted r^2^
Model	10	16.08	<0.0001	5572.43		0.54
Error	130			346.54		
Total	141			106694		

Markers are given in order of inclusion in the trait model according to GLM Select. AIC is the Akaike Information Criterion and estimates the goodness of fit for the model. Significance of the association between marker and trait is demonstrated by F and p values. The t-value estimates the magnitude of the effect; a positive score indicates Mo17 donated the superior allele. Marker locations are reported using IBM v1 coordinates (chromosome; position). Summary statistics for the 10-factor model are presented below.

In parallel to our work on grain Fe nutrition, we have also collected elemental concentration data for other grain components (Hoekenga, Rutzke and Kochian, unpublished data). It has been reported that several other mineral elements may influence Fe bioavailability in positive or negative ways, by competing with Fe uptake into intestinal cells [28], [29]. Pearson's correlation analysis was performed on FeGB and FeGC with grain mass and Ca, P and Zn concentrations for the NY03 data (Table 5). There was a significant, positive correlation between the levels of all of these mineral elements, Ca, Fe, P and Zn, ranging from r  =  +0.206 to +0.511. It is not obvious what factor would coordinately control mineral nutrient densities for all four of these minerals. This correlation between mineral nutrients did not appear to be a function of grain mass; negative correlations exist between Ca, Fe, P and Zn grain concentration and grain mass, while only Ca and P were significant. Grain P concentration was the only parameter that correlated with FeGB, although this effect is small (r^2^<0.04). Grain Fe concentration and bioavailability were not significantly correlated, which is not surprising given the general lack of agreement between FeGB and FeGC QTL locations. This suggests that FeGB and FeGC are under the regulation of different major determinants.

**Table 5:** Correlation analysis of grain nutrients and mass.

	Ca grain concentration	Fe grain concentration	P grain concentration	Zn grain concentration	Fe grain bioavailability
**Fe grain concentration**	**0.206/0.002**				
**P grain concentration**	**0.43/<0.001**	**0.417/<0.001**			
**Zn grain concentration**	**0.213/0.001**	**0.439/<0.001**	**0.511/<0.001**		
**Fe grain bioavailability**	*-0.03/0.725*	*0.101/0.234*	**-0.191/0.024**	*0.076/0.374*	
**Grain mass**	**-0.254/<0.001**	*-0.119/0.072*	**-0.174/0.008**	*-0.045/0.496*	*0.147/0.079*

Pearson's correlation coefficient (left) and p-value (right) are reported for each correlation. Bold entries indicate significant correlations; italic entries indicate non-significant correlations from the NY03 dataset.

We analyzed 23 RI lines selected from the extremes of grain Fe bioavailability, along with several from near the population median, to determine if a correlation existed between phytate concentration and grain Fe bioavailability (Table 6). Phytate is widely regarded in the literature as the major anti-nutrient compound that limits Fe bioavailability in grain crops [12]. Pearson's correlation analysis indicated there was a small, weak negative correlation between FeGB and phytate concentration (r  =  −0.19; N.S.). However, grain phytate concentration among the high, medium and low FeGB RI samples were not different by one-way ANOVA (Table 6). These data suggest that phytate was not a significant determinant for differences in Fe bioavailability in the IBM RI population, or at least in the sub-sample of the RI population tested.

**Table 6:** Comparison between FeGB and phytate content (NY 03).

FeGB level (# RIL tested)	Average ferritin ng total protein mg^−1^ (± sd)	Average phytate µmoles g^−1^ (± sd)
High (5)	54.6±2.3	9.2±2.8
Moderate (9)	27.0±1.2	9.3±1.6
Low (9)	12.2±2.2	9.9±1.0

To validate the FeGB QTL model, we conducted a co-segregation analysis using backcross-derived families segregating for three of the major QTL. Molecular markers were used to assess which individuals would be worthwhile to phenotype from a collection of derivatives of IBM RI lines that had been previously initiated from 12 different RI lines backcrossed to both parents. Nine families of BC_2_S_2_ or BC_3_S_3_ individuals from this collection were genotyped with eight simple sequence repeat markers that spanned three QTL containing intervals (FeGB-3.1, FeGB-6.1 and FeGB-9.1) that were detected by both the conservative (CIM) and permissive (GLM Select) analyses. From the marker analysis, we identified 37 individuals from the NY06 field season that were self-pollinated and then analyzed using the Caco-2 bioassay. Most of the backcross-derived individuals selected for phenotypic analysis contained all three superior or inferior alleles, to maximize the potential degree of difference between samples. Three of the molecular markers tested gave highly significant associations with FeGB, one for each of the three chromosomal regions (Table 7). The superior alleles detected in the backcross-derived lines were the same as those originally detected in the RI lines, supporting the original QTL analysis. Thus, we were able to select individuals based solely on molecular marker information out of segregating populations and correctly predict the FeGB nutritional quality of those individuals. These results not only affirm that three QTL for FeGB exist on chromosome 3, 6 and 9 of the maize genome but also that marker assisted selection can efficiently enhance FeGB.

**Table 7:** Marker co-segregation analysis of BC_2_S_3_ and BC_3_S_4_ derived families (NY 06).

Marker	Location	Mean FeGB ± sd for B73 allele	Mean FeGB ± sd for Mo17 allele	F-score	p-value
UMC1742	3; 188	28.3±1.1	33.7±1.2	9.81	0.0001
BNLG1641	6; 76	32.1±1.2	28.3±1.4	6.50	0.0022
UMC2343	9; ∼365	31.4±1.5	27.0±1.1	5.22	0.007

Average ferritin production values (ng ferritin mg^−1^ total protein) from Caco2 bioassays are reported for homozygous BC_2_S_3_ or BC_3_S_4_ individuals from the NY 2006 field season. Correlation of allelic state with iron bioavailability was assessed using one-way ANOVA; F-scores and p-values report the significance of differences. Location refers to (chromosome; position) in IBM v1 cM.

### Derivation of new inbred lines with altered FeGB quality

**Figure 3:**
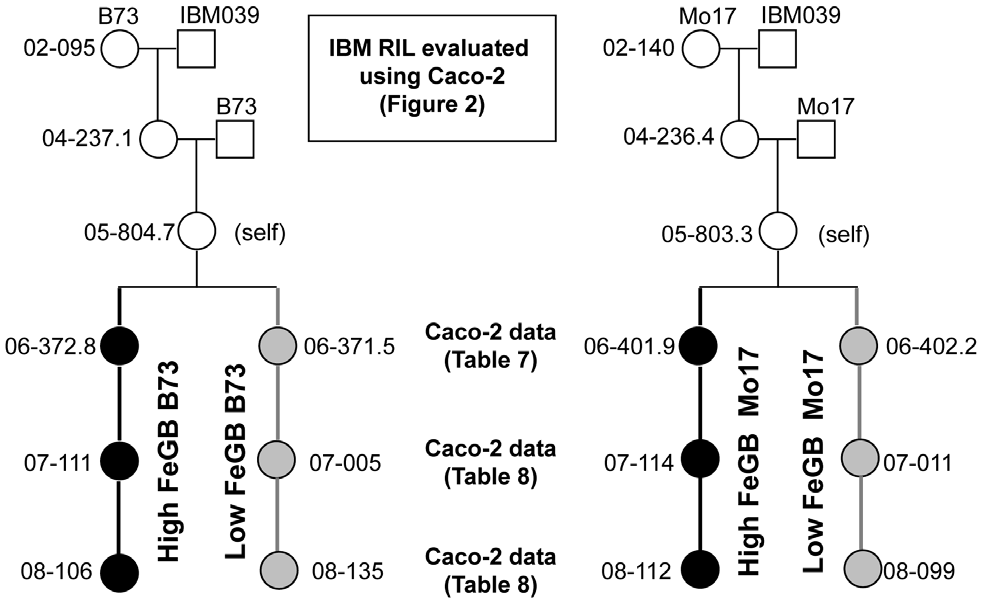
Pedigree for sister derived lines with altered FeGB qualities. High and low FeGB derivatives were generated from IBM RI Line #039 using backcrossing to both B73 and Mo17 parents. Circles denote maternal parents and squares are paternal parents, where the numbers that appear next to the circles or square refer to the field entry (e.g. 02-095 indicates NY2002 row 095). Caco-2 phenotyping was utilized at four points in this process: for the evaluation of RI lines (Figure 2), to validate the QTL model (Table 7), and to assist derivation of new inbreds (Table 8). The backcrossing program was initiated three years before Caco-2 phenotyping of the RI lines took place. Individuals with altered FeGB qualities are denoted with filled black circles (high FeGB) or gray circles (low FeGB). While not shown here, lines have been advanced to the BC_2_S_6_ (highly inbred) generation in the NY2010 field season, with an additional round of Caco-2 phenotyping occurring in early 2011.

Based upon the molecular marker and phenotypic characterizations, selections were made from the backcross derivatives of IBM RI line #039 to generate new inbred lines with altered nutritional qualities (Figure 3). While backcross derivatives from nine different IBM RI lines were screened, the derivatives from IBM RI line #039 gave especially reproducible results. Seeds were chosen from single BC_2_S_3_ individuals to represent four new genotypes: high FeGB B73, low FeGB B73, high FeGB Mo17 and low FeGB Mo17. These four genotypes were sent to collaborators for evaluation at six sites beyond our regular NY location. Self-pollinated seeds were generated and analyzed by the Caco-2 bioassay (Table 8) and ICP (Table 9). We hypothesized that significant differences would exist between high and low seed bioavailable Fe sister derived lines at many or all locations where they were grown. Beyond NY, high and low sister derived lines were significantly different when grown in Ames IA, Urbana IL, Puerto Vallarta MX, and Clayton NC. This comes with the caveats that only the B73 sister lines were grown at Puerto Vallarta and that the Mo17 sister lines did not produce a statistically significant outcome at Clayton NC, although the trend was in the expected direction. Likewise, while samples from State College PA were not significantly different, the trends were in the expected directions. No differences were observed from samples from Columbia MO for FeGB (Table 8). While this experiment was limited in scale, we conclude that the enhanced FeGB quality identified in NY grown materials is effective at locations outside of NY. Based on our prior experience, we did not expect to see significant differences in FeGC between the sister lines. This hypothesis was supported by results from MO, MX, and NC (Table 9). However, significant differences in FeGC did exist between samples grown at IA, IL, and PA. Given the lack of consistency between rankings, it is not clear what factors might have been at work in influencing FeGC.

**Table 8:** Multi-site evaluation of FeGB in derived lines.

Site-Year	High B73	Low B73	High Mo17	Low Mo17	F-score
IA-2008	0.671 b	0.459 c	0.813 a	0.597 b	15.727
IL-2007	1.573 a	0.974 b	1.411 a	1.055 b	12.370
*MO-2008*	*1.099 b*	*0.974 b*	*1.582 a*	*1.410 a*	*17.672*
MX-2008	1.100 a	0.782 b	n.d.	n.d.	40.916
NC-2008	0.809 a	0.573 c	0.740 ab	0.671 b	7.961
NY-2007	0.923 a	0.791 b	0.870 ab	0.600 c	12.524
NY-2008	1.208 a	0.379 b	1.238 a	0.490 b	37.247
*PA-2008*	*0.495 ab*	*0.344 b*	*0.598 a*	*0.448 ab*	*3.762*

Contrasting BC_2_S_4_ derivatives from the IBM039 RI line were grown on 8 plots over 2 years, to evaluate the heritability and penetrance of the high FeGB effect across multiple environments. ANOVA were used to assess whether pairs of related high and low-nutritional value derivatives were significantly different and are denoted by letter. Comparisons were made within sites only, where trait data are expressed as a percentage of the control variety from the Caco-2 bioassay. Locations where significant differences were not observed according to our hypotheses appear in italic type.

**Table 9:** Multi-site evaluation of FeGC in derived lines.

Site-Year	High B73	Low B73	High Mo17	Low Mo17	F-score
IA-2008	25.06 a	17.48 c	23.14 b	23.25 b	32.29
IL-2007	23.54 b	27.21 a	25.18 ab	23.63 b	4.23
*MO-2008*	*24.03 ab*	*24.33 ab*	*25.87 a*	*20.82 b*	*1.80 (ns)*
*MX-2008*	*20.51 a*	*21.42 a*	*n.d.*	*n.d.*	*3.61 (ns)*
*NC-2008*	*24.55 a*	*24.10 a*	*22.69 a*	*23.78 a*	*0.25 (ns)*
*NY-2007*	*19.90 a*	*20.65 a*	*21.16 a*	*26.91 a*	*1.77 (ns)*
NY-2008	23.22 a	18.00 b	23.84 a	22.87 a	9.37
PA-2008	20.52 b	19.93 b	24.41 a	21.32 b	16.32

FeGC was evaluated among accessions grown in 2007 and 2008. Comparisons were made within sites using ANOVA, where trait data reported are entry averages for grain iron concentration in µg g^−1^ DW. Locations where significant differences were not observed appear in italic type.

## Discussion

The objective of this study was to estimate the genetic component(s) underlying maize grain Fe nutrition. We were able to identify multiple loci that influence grain Fe concentration and bioavailability, and demonstrated these were heritable across multiple years. While these loci may not explain a majority of the differences observed, they show promise that genetic analysis will be useful to dissect questions in maize relating to human Fe nutrition. These experiments provide entry points into these nutritional processes at the genetic and ultimately molecular levels. These experiments also serve as a demonstration of the utility of a forward genetic approach to dissect grain Fe nutrition, as the QTL described here can improve Fe concentration and bioavailability to a degree comparable with existing transgenic or reverse genetic approaches.

Biofortification, or the nutritional enhancement of foods via the direct improvement of the crops that derive them, has been a topic of great interest in recent years [30]–[34]. Until very recently, the focus of this discussion has been upon the possible approaches and potential impacts, but relatively little research has been performed with regard to elemental micronutrients. The first experimental studies in this area for maize were largely germplasm surveys for micronutrient concentration [35], [36]. While these studies demonstrate that the genetic potential for maize improvement exists, neither study enhanced our understanding of the nature of grain micronutrient density, *per se*. QTL analysis has been applied to mineral nutrient density in *Arabidopsis thaliana* and *Phaseolus vulgaris*; unfortunately, none of these studies address the issue of nutrient bioavailability [37]–[40]. Thus, there is a clear gap in the literature that the research presented in this study aims to begin to fill.

Transgenic approaches to grain Fe biofortification have been attempted for rice, wheat and maize [24], [41]. In these studies an approximately 2-fold increase in grain Fe concentration was observed, presumably by increasing the metabolic sink in the grain via over-expression of a soybean or common bean ferritin protein [41]. Attempts have also been made to increase the bioavailable fraction of grain Fe with transgenic expression of phytase, an enzyme that catalyzes the breakdown of phytate. In maize, this strategy increased bioavailable Fe in grains by approximately 2-fold in the best transgenic event, which translated to an increase of 20 ng ferritin produced mg^-1^ total protein in the Caco-2 bioassay [24]. Transgenic approaches for biofortification suffer from two possible limitations. First, it is impractical to use transgenic plants as a forward genetic tool; the present bioassay for Fe bioavailability is somewhat restricted in scale – analysis of hundreds rather than thousands of samples are the present level of practicality. It is also unlikely that screening mutagenized populations for mutants that alter Fe bioavailability is possible, given the number of random mutants necessary for a saturating screen. Thus, transgenic plants are likely only useful in reverse genetic experiments, where a particular putative modifier of Fe bioavailability or nutrition is being tested. Second, societal acceptance for transgenically improved crops does not exist in every quarter, such that relying solely upon transgenic solutions will have limited application. On the other hand, a QTL-based approach for genetic discovery, within a larger, interdisciplinary research scheme, overcomes these limitations. QTL analysis can effectively survey the genetic diversity present in a mapping population using hundreds of bioassays, to build a genetic model for the complexity of the trait of interest. The information gained from this analysis can then be utilized for either transgenic or traditional crop improvement.

Our genetic analysis of the IBM RI set identified three modest sized QTL that contributed to approximately one-quarter of the variation in grain Fe concentration (Table 2). However, the estimated heritability for this trait is three times as large, which indicates that grain Fe concentration is under the influence of many QTL that are too small to detect using the methods we employed (Table 1). The analysis of variance also made clear that local environment plays a strong role in influencing grain Fe concentration. The field plots used in NY03 and NY05 fell in different, distinct soil types: the maize from NY03 was grown on a Lima Silt Loam (alfisol) with an average maize yield of 120 bushels acre^−1^, while maize from NY05 were grown on a Kendaia Silt Loam (inceptisol) with only 95 bushels acre^−1^ average productivity [42]. The same agronomic management practices were used for both seasons in NY and shared similar weather, typical to NY. In comparison, NC05 was planted on a Norfolk Loamy Sand (ultisol) soil, with average maize yields of 106 bushels acre^−1^, where NC enjoys higher average day and night temperatures, shorter day length and somewhat less rain than NY. We observed that there was a higher degree of correlation for FeCG between NC05 and NY05 than between NY05 and NY03, which suggests that soil properties may play a stronger role than weather or agronomic practices to influence FeCG. Future studies will require a far better understanding of local soil conditions and properties to better describe the environmental factors that influence grain Fe concentration. While grain Fe concentration could be a target for biofortification efforts, substantial progress using conventional plant breeding may be difficult to achieve using marker assisted selection and. We predict that more comprehensive technologies such as genomic selection, which are more effective at accounting for and combining many small effect QTL, may be necessary to enhance FeGC by plant breeding [43].

On the other hand, our genetic analysis of grain Fe bioavailability identified multiple putative QTL. Using a conservative approach, three modest QTL were identified that explained a quarter of variation observed in bioavailable Fe, similar to that seen for Fe concentration (Table 3). However, the single marker analysis found 7 additional significant associations, explaining 54% of the phenotypic variance observed in FeGB (Table 4). As we can build a more comprehensive genetic model to explain variation in FeGB, this suggests that FeGB is a less genetically complex trait than FeGC and thus more tractable. The lack of a strong, positive correlation between FeGC and FeGB also suggests that FeGB is the far more valuable trait to evaluate, although FeGB is more difficult to phenotype given the limitations and requirements of the Caco-2 bioassay (Table 5). We have demonstrated the efficacy of marker assisted selection for FeGB in the development of our sister derived lines (Tables 7 & 8), such that moving the elite alleles detected in the IBM population into other germplasm can be easily accomplished using genotype based methods. Caco-2 bioassay based phenotyping could be reserved for later stages in a breeding program, to confirm the value of selections rather than as a selection tool itself.

Near isogenic lines (NILs) are commonly used tools to dissect QTL [44]. NILs represent very highly related varieties that differ at perhaps a single QTL, and are useful to dissect QTL function and identity. We were concerned that isolating single QTL in new varieties would not create large enough changes in FeGB to be detected through our process of using field-grown plants and a bioassay for phenotyping. Thus, we chose to combine the three QTL detected using composite interval mapping in new varieties, derived by backcrossing particular IBM RI lines to either B73 or Mo17. This strategy was clearly successful from the perspective of producing new varieties with reproducible differences in FeGB (Tables 7 & 8). In the NY10 field, these lines have been advanced to the BC_2_S_6_ generation and evaluated using the Caco-2 bioassay (data not shown). Our sister derived lines should now be stable due to the high degree of inbreeding. Our decision to pursue both high and low FeGB selections into both the B73 and Mo17 parental backgrounds was made for two reasons. First, the high and low selections share three generations of single seed descent and are at least 87.5% genetically identical to each other. While these are not isogenic stocks, these sister derived lines do represent an improvement over using IBM RI lines with regards to normalizing the effect of the remainder of their genomes. These new inbreds make excellent targets for detailed metabolomic and genomic studies, perhaps using next generation sequencing tools, to more fully describe how they have altered nutritional qualities. Should they be necessary, true NILs could be constructed by backcrossing our new inbreds to their recurrent parents and then selecting out individuals with one, two or three QTL combinations for analysis of individual genes. Second, B73 and Mo17 are known to have excellent combining ability, where B73 × Mo17 was a widely commercialized hybrid variety used by many North American seed companies through the 1970s and 1980s. Making hybrids among the high FeGB and the low FeGB sister derived lines could create largely identical hybrids with altered FeGB quality, which would facilitate both agronomic studies and animal feeding trials by taking advantage of heterosis to boost grain production.

While it has yet to be established whether the amount of variation in FeGB present in the IBM RI population or the derived inbred lines is sufficiently large to be immediately useful for biofortification, we have demonstrated the utility of a QTL/Caco-2 based strategy to investigate FeGB. These new genetic tools in maize should rapidly permit animal and human nutritional studies, whether single meal feeding or longer term studies, to more thoroughly assess the impact of our work. We selected the IBM RI panel for grain Fe nutrition testing based upon our prior experience with this mapping population and the wealth of genetic and genomic resources available. It is certainly possible that other RI populations possess broader phenotypic ranges or more simple genetics for FeGB or FeGC than those observed here. It should be a profitable strategy to survey additional RI populations, using both the analytical chemistry and bioassay methods utilized here, to identify additional determinants for grain Fe nutritional quality. For example, phenotyping the Nested Association Mapping Panel of maize would be extremely worthwhile and powerful experiment, given the exceptional capacity of that 5,000 RI line population to resolve QTL [45], [46]. In parallel, once the genes that underlie that major FeGB QTL are identified, it should be possible to identify the natural variants that already exist in breeding populations, which would enable Fe biofortification efforts around the world using conventional breeding techniques. However, once the genes and gene products have been identified that enhance FeGB in our study system, it should also be possible to enhance FeGB by transgenic means. By either mechanism, it should soon be possible to biofortify maize and other staple food crops with additional bioavailable iron.

## Materials and Methods

Unless otherwise stated, all chemicals, enzymes and hormones were obtained from Sigma Chemical Company (St. Louis, Mo).

### Plant materials and field site details

The IBM RI population was received from the Maize Genetics Cooperation Stock Center (Urbana, IL) and grown at research farms owned by Cornell University and North Carolina State University. Fields were planted at the Musgrave Farm (Aurora, NY) in the summers of 2003, 2005, 2006, 2007 and 2008 at the Central Crops Research Station (Clayton, NC) in 2005. The plots used in 2003, 2006, and 2007 on the Musgrave Farm had a Lima Silt Loam (alfisol) soil, with average yield for maize of 120 bushels acre^−1^ and water extractable soil pH of 6.7, in 2005 were on a Kendaia Silt Loam (inceptisol), with an average yield for maize of 95 bushels acre^−1^, and a water extractable soil pH of 6.5, and in 2008 were on a Honeoye Silt Loam (alfisol), with an average yield for maize of 130 bushels acre-1, and a water extractable soil pH of 6.1, while the Central Crops Research Station plots had a Norfolk Loamy Sand (ultisol) soil, with average maize yields of 106 bushels acre^−1^ and water extractable soil pH of 4.8 according to the Web Soils Survey of the National Resource Conservation Service (http://websoilsurvey.nrcs.usda.gov) [42]. In 2003, single randomized, partial blocks of the RI population were used for this study (n = 232). A subset of RILs was used for the Caco-2 bioassay described below (n = 145). Pioneer Hi-Bred (a DuPont Company) donated untreated grain from 5 hybrid varieties for use as possible controls in the Caco-2 bioassays. In 2005, replicated, randomized partial blocks were grown in NY and NC and used for the mineral analysis (NY n = 257, 3 replicate blocks; NC n = 274, 2 replicate blocks).

Sister derived inbred lines were developed from backcross (BC) derivatives of 12 IBM RILs (ie. 24 sets of families). The BC line project was initiated as a component of a National Science Foundation Plant Genome Research project on aluminum stress tolerance in maize roots. Fortuitously, several of the derivative families were segregating for markers linked to the grain Fe bioavailability QTL and thus of use to this study. In 2006, representatives of 9 of the 24 BC_2_S_2_ and BC_3_S_3_ families were planted in randomized blocks, genotyped using SSR marker analysis and all individuals were self-pollinated. Of these, 37 BC_2_S_3_ and BC_3_S_4_ ears were selected for Caco-2 bioassay phenotyping to validate the FeGB QTL models. In 2007 and 2008, confirmed high FeGB and low FeGB sister lines were grown to increase the degree of inbreeding and evaluate using the Caco-2 bioassay. Seeds from single BC_2_S_3_ sources were used for evaluation at Aurora NY, Ames IA, Urbana IL, Columbia MO, Clayton NC, and State College PA. Seeds for the trial at Puerto Vallarta MX were generated at Urbana IL. Collaborators generated self-pollinated seed that were evaluated using ICP and the Caco-2 bioassay as described below.

### Mineral analysis

Mineral analysis of the samples was conducted by inductively coupled plasma-emission spectroscopy (ICAP; ICAP model 61E Trace Analyzer; Thermo Jarrell Ash Corporation, Waltham MA). Twenty-five grains were ground to fine powder using a coffee mill (Capresso Inc.), where RI lines were sampled once and parents were sampled six times (i.e. 6×25 grains). 1 g samples of ground maize were weighed into borosilicate glass test tubes and chemically digested using 1ml of 100% HNO_3_ at 120°C, followed by drying the samples completely. Further addition of 1 ml of 100% HNO_3_ was carried out at 150°C until the residue was light brown to yellow in color. Then 1 ml of HNO_3_: HClO_4_ at 1∶1 volume ratio was added and the temperature increased to 180°C for 2 hours and then to 240°C until the digested samples were dry. Samples were then resuspended in 5% (v/v) HNO_3_ before analysis on the ICP.

### Quantifying Grain Fe bioavailability

#### Sample preparation

Kernels (50g) were sorted to remove any debris and then placed in an acid washed beaker and covered with 2 volumes of 18 MΩ water. Kernels were then autoclaved at 121°C and at a pressure of 115 kPa for 40 min, allowed to cool at room temperature and then frozen overnight at −20°C. Samples were then freeze dried at 100 millTorr and a temperature of −50°C for 7 days, ground to a fine powder with a coffee mill (90 sec) and stored in acid washed, plastic specimen containers with tight fitting lids at 25°C (Laboratory Product Sales, Rochester NY). Samples from the commercial hybrid maize were prepared in an identical manner and used as controls for the each of the bioassays.

#### Quantifying Fe availability

The test for Fe availability of maize grain Fe was carried out using the Caco-2 in vitro digestion method as described by [14]. In this model, cell ferritin formation in response to Fe uptake is used as a marker of Fe bioavailability. Ascorbic acid (Asc) was added to enhance Fe bioavailability using a 20∶1 Asc:Fe molar ratio, based upon highest FeGC observed. Once mixed, 0.25 mL of pepsin solution (trace mineral free) was added. Total cellular protein was determined in the lysates by the BioRad DC Protein Assay Kit (BioRad, Richmond, CA). Ferritin content was determined using a one-stage, two-site immunoradiometric assay (FER-Fe^2+^ Ferritin Assay, RAMCO Laboratories, Houston TX) (Glahn et al., 2002). Ferritin contents were normalized to total cellular protein concentrations; ferritin values for each RI sample were then expressed as a percentage of the control maize (commercial hybrid) sample to standardize the results of the bioassays.

### Phytate analysis

Phytate was analyzed using acidic extraction of the maize grain meal, followed by liquid chromatography [47]. Samples were analyzed with a Dionex Liquid Chromatograph System (Dionex Corp., Sunnyvale, CA) using PO_4_ and phytate standards (IP_5_ and IP_6_) dissolved in 0.125% (v/v) H_2_SO_4_. The results are expressed as µmole of phytate per gram (DW).

### Data Analysis

Basic statistical (one-way ANOVA, Pearson's Correlation) analyses were performed using SAS v 9.1.3 for Windows (www.sas.com, Cary NC) or JMP v8 for Macintosh. Genetic marker information for the IBM population was downloaded from http://www.maizegdb.org/qtl-data.php (verified 2/11/11). A genetic map with 1,338 markers and overall length of 6,243 cM in ten linkage groups was used for all analyses. QTL searches were conducted on best linear unbiased predictors (BLUP) of FeGC, estimated from the ANOVA for the six site/year replicate data sets, balanced by year and site. Broad sense heritability (h^2^_b_) was estimated from the mean sum of squares calculated from the ANOVA table, with

h^2^_b_  =  MS_between RIL_/(MS_withinRIL_ + MS_betweenRIL_).

A trait with no variance within repeated measurements of the RILs would have an h^2^_b_  =  1 and thus be completely heritable.

QTL searches for FeGB were conducted on Caco-2 bioassay values of ferritin protein production standardized according to average response of the Caco-2 cells to a control variety of maize. QTL analysis by composite interval mapping was conducted using QTL Cartographer v 2.5 for Windows, with forward and backward regression (window  = 5 cM, step  = 2 cM, p(in/out)  = 0.01) [48]. Summary models were estimated using the Multiple Interval Mapping procedure in QTL Cartographer. QTL analysis by single marker analysis was conducted using the GLM Select procedure in SAS.

### Molecular Marker Analysis

Linkage analysis was conducted using standard methodologies for simple sequence repeat markers resolved on 4% agarose gels. Primer sequences were selected from the Maize Genetics and Genomics Database (http://www.maizegdb.org) [49].
